# The favorable outcome of Bernese periacetabular osteotomy for the hip osteoarthritis in multiple epiphyseal dysplasia

**DOI:** 10.1186/s13023-023-02920-1

**Published:** 2023-10-30

**Authors:** Yao-Yuan Chang, Chia-Che Lee, Sheng-Chieh Lin, Ken N. Kuo, Jia-Feng Chang, Kuan-Wen Wu, Ting-Ming Wang

**Affiliations:** 1 Departments of Orthopedic Surgery, National Taiwan University Hospital, No.7, Chung Shan S. Rd., Taipei City, 100 Taiwan; 2 Department of Orthopaedic Surgery, Chung Shan Medical University Hospital, Taichung, 402 Taiwan; 3 Institute of Medicine, Chung Shan Medical University, Taichung, 402 Taiwan; 4 Cochrane Taiwan, Taipei Medical University, Taipei, 110 Taiwan; 5 Department of Internal Medicine, En Chu Kong Hospital, New Taipei City, 237 Taiwan; 6 TMU Research Center of Urology and Kidney (TMU-RCUK), Taipei Medical University, Taipei, 110 Taiwan

**Keywords:** Multiple epiphyseal dysplasia, Bernese periacetabular osteotomy, Hip osteoarthritis and hip dysplasia

## Abstract

**Background:**

Multiple epiphyseal dysplasia (MED) is a rare congenital bone dysplasia. Patients with MED develop secondary hip osteoarthritis as early as the third to the fourth decade. Currently, there is no consensus on the prevention of the progressive hip osteoarthritis secondary to MED. The Bernese periacetabular osteotomy (PAO) is a joint-preserving surgery to reshape acetabulum and extend femoral head coverage. However, there is no documentary evidence for the effect of the procedure on MED hips.

**Patients and methods:**

We analyzed the preliminary outcomes following the Bernese PAO in 6 MED hips. The average age at the time of surgery was 14.3 years (range from 11.4 to 17.2 years). For our study interest of time efficiency, radiographic parameters were analyzed preoperatively and 1 year postoperatively. The hip function was evaluated by the Harris Hip Score (HHS) before and after surgery.

**Results:**

The mean follow-up time was 1.7 years. The mean lateral center–edge angle increased from 3.8° to 47.1° (*p* = 0.02), anterior center–edge angle increased from 7.3° to 35.1° (*p* = 0.02), and acetabulum index decreased from 27.8° to 14.6° (*p* = 0.04). The femoral head coverage ratio increased from 66.8% to 100% (*p* = 0.02). The post-operative anteroposterior pelvic radiograph demonstrated all preoperative broken Shenton lines were reversed. The mean HHS improved from 67.3 to 86.7 (*p* = 0.05).

**Conclusion:**

Bernese PAO is a feasible treatment for hip disorders in MED patients. It reshapes acetabular and femoral morphology efficiently. In our study, the preliminary results showed the procedure not only improved radiographic outcomes but also hip function.

## Introduction

Multiple epiphyseal dysplasia (MED) is a rare, congenital bone dysplasia characterized by abnormal ossification of multiple epiphyses [1]. It affects 10 per 100,000 individuals worldwide [2]. The clinical presentations include short stature, angular or rotational deformity of the extremities, intermittent joint pain, and/or precocious osteoarthritis. Weight-bearing joints are prone to be affected, especially the hip joints [3]. Mäkitie et al. showed all patients with MED developed abnormal ossification in the proximal femoral epiphyses before skeletal maturity in a study of 12 patients [4]. Patients with MED were doomed to suffer from secondary hip osteoarthritis as early as the third to the fourth decade [5]. The information on the results of total hip arthroplasty in patients with MED was scarce. Ramaswamy et al. reported 16 hips with osteoarthritis secondary to MED received total hip arthroplasty. The mean age at the time of surgery was in the third to fourth decade of life. Ten hips required revision at a mean time of 12.5 years 3. To prevent secondary hip osteoarthritis, the potential therapeutic options include conservative treatments (a decrease in weight bearing with assistive device, controlling body weight and physical training to increase muscular strength) and acetabular osteotomy. Chiari Osteotomy, Steel triple innominate osteotomy and Dega osteotomy have been investigated to redirect or reshape the acetabulum in the management for MED patients of different ages [6–8]. The short-term results of prior studies were impressive. Currently, there is no consensus on the prevention of the progressive hip osteoarthritis secondary to MED [1].

The Bernese periacetabular osteotomy (PAO) is a procedure to reshape acetabulum and extend coverage of the femoral head. The most frequent surgical indication is symptomatic acetabular dysplasia in adolescents or young adults with correctable deformity and limited range of motion. The contraindications include advanced osteoarthritis and irreducible incongruity of the hip joint [9]. Currently, the Bernese PAO has been recognized as an effective surgery to prevent secondary hip osteoarthritis in patients with hip dysplasia. The 10-year cumulative hip event-free rate is 80–90% [10]. Although there are acetabular dysplasia and hip joint incongruity in patients with MED, most of hip joint subluxation can be reduced with internal rotation, flexion, and abduction. According to the above documentary evidences, we hypothesized Bernese PAO might improve the osseous stability of these hip joints.

Therefore, the objectives were to assess the short-time surgical outcomes in MHD patients undergoing Bernese PAO.

## Materials and methods

### Study participants and surgical procedure

This retrospective study includes consecutive patients with diagnosis of MED between January 2015 and December 2020. The diagnosis was based on clinical presentations and plain radiographs by pediatric geneticists and orthopaedic surgeons. The clinic assessment of preoperative and postoperative medical records and radiographs were available for the study. Symptomatic MED patients received the Bernese PAO with minimum of one-year follow-up were enrolled in the study. Indications for surgery included limp, a minimum of conservative treatment of intolerable hip pain for more than 6 months, lateral center–edge angle (LCEA) less than 20 degrees and Tönnis grade less than 2. All patients had three dimensional computed tomography (3D-CT) scan of hip joints before the surgery to check the hip congruity and femoral head coverage. Only reducible hip joints without hinge abduction in frog leg radiographs met the criteria for surgical intervention. Accordingly, a total of 6 hips in 3 patients with MED fulfilled the criteria of inclusion.

The surgical incision was performed via Smith-Petersen incision about 6–8 cm which is smaller than the originally described [11]. Osteotomies of the anterior portion of ischium, the superior pubic ramus, the ilium, and then along the posterior column were performed via angled bone chisel. After osteotomies, the fragments were fixed with threaded pins, or cannulated screws. At the end of our procedure, all patients were checked for hip internal rotation of 20 degrees with hip flexion to 90 degrees without difficulty.

We conducted surgeries on both hips of a patient in two separate procedures, with an average time interval of 104 days before the contralateral hip underwent surgery.

### Post-operative protocol

After operation, rehabilitation program started as soon as possible. With physical therapist’s assistance, the patients started toe-touch weight bearing ambulation. The weight bearing as tolerated with crutch assistance began one month postoperatively. The patients progressed to full weight bearing without assistance six months postoperatively. All patients were expected to return to normal activities of daily living in one year.

### Imaging evaluation

Radiographic parameters were analyzed preoperatively and one year postoperatively. The radiographic parameters included femoral head coverage ratio, LCEA, acetabulum index (AI), central head distance, and Shenton’s line on the AP view. Anterior center-edge angles (ACEA) were recorded on the false-profile view [12]. The leg length was measured on the scanogram.

### Clinical evaluation

Hip function was evaluated by the Harris Hip Score (HHS) [13] before surgery and one year postoperatively. Questionnaires for pain and functional domains of HHS were translated in Mandarin for better understanding. In addition, pain was also evaluated by the Visual Analogue Scale (VAS) with 5 being the most severe.

### Statistical analysis

Pre- and postoperative radiographic parameters and HHS scores were compared using a Mann–Whitney *U* test. Statistically significance was defined as *p* < 0.05. The statistical analysis was performed by SPSS version 22.0 (SPSS Inc. Chicago, USA).

## Results

### Demographics

Three female MED patients who had index operation at our institution with a minimum of one-year follow-up were included. Triradiate cartilages were closed in all plain pelvis radiographs. All 3 patients received bilateral Bernese PAO in stages. None of them received other surgical interventions before Bernese PAO. The mean age at the surgery was 14.3 years (range from 11.4 to 17.2 years) and the mean body mass index was 20.2 kg/m^2^ (range from 17.5 kg/m^2^ to 21.4 kg/m^2^). The mean follow-up time was 1.7 years (range from 1.21 to 2.98 years) (Table 1).

**Table 1:** Demographic features of the patients with MED included in the study

Patient No	Side	Age	BMI	Pain score	Tönnis grade	Follow-up (y)
1	Right	15.6	21.4	3	1	1.21
1	Left	15.0	21.2	4	1	1.81
2	Right	11.4	17.5	4	1	2.38
2	Left	10.8	18.4	3	1	2.98
3	Right	17.2	21.2	4	1	1.51
3	Left	16.6	21.2	4	1	2.11

### Radiographic results

Preoperative 3D-CT showed all 3 patients had incongruent hip joint surface and global dysplasia at acetabulum with variable deficiencies in coverage of femoral head.

**Fig. 1:**
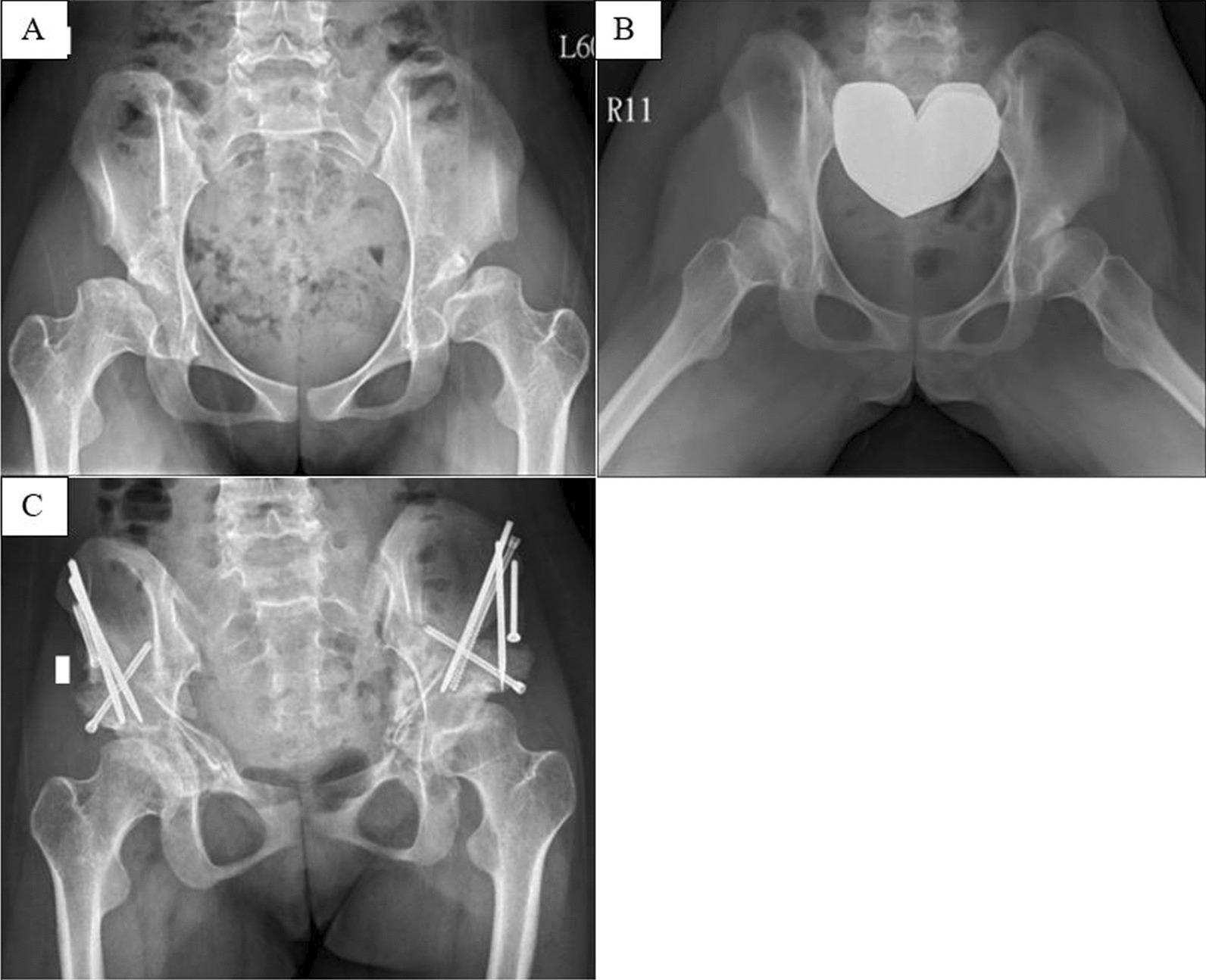
The comparisons between preoperative and postoperative outcomes in the anterior–posterior pelvic radiograph

The mean LCEA increased from 3.8$^\circ$ (range from − 23.1$^\circ$ to 14.4$^\circ$) to 47.1$^\circ$ (range from 37.7$^\circ$ to 55.4$^\circ$) (*p* = 0.02), ACEA increased from 7.3$^\circ$ (range from −8.3$^\circ$ to 16.2$^\circ$) to 35.1$^\circ$(range from 29.9$^\circ$ to 41.0$^\circ$) (*p* = 0.02), and AI decreased from 27.8$^\circ$ (range from 24.9$^\circ$ to 31.8$^\circ$) to 14.6$^\circ$ (range from 5.8$^\circ$ to 21$^\circ$) (*p* = 0.04). The femoral head coverage ratio increased from 66.8% (range from $46\mathrm{\%}$ to 79%) to 100% (range from $100\mathrm{\%}$ to 100%) (*p* = 0.02). Femoral head medialization was evident by the decreasing central head distance from 86.7 mm to 82.7 mm. Nonetheless, it was statistical non-significance from the small sample size of this rare disease (*p* = 0.699). The leg length had no obvious difference during the short-term follow-up. All broken Shenton lines were restored following surgery (Table 2). An example of the patient No.2 radiographs is shown as Fig. 1.

**Table 2:** The preoperative and postoperative comparisons of radiographic parameters in patients with MED following the Bernese PAO

Parameters	Pre-operation		Post-operation		p-value
	Mean	Range	Mean	Range	
LCEA (deg.)	3.8	− 23.1 to 14.4	47.1	37.7 to 55.4	0.02
ACEA (deg.)	7.3	− 8.3 to 16.2	35.1	29.9 to 41.0	0.02
AI (deg.)	27.8	24.9 to 31.8	14.6	5.8 to 21	0.04
Femoral head coverage (%)	66.8	46 to 79	100	100 to 100	0.02
Central head distance (mm)	86.7	83 to 91	82.7	70 to 88	0.699
Leg length (cm)	63.5	56.5 to 69.6	62.8	53.5 to 69.7	0.463
Shenton line	Broken		Intact		–

All osteotomies achieved bony union at 6 months following surgery. Neither delayed union nor nonunion was observed. No progression of hip osteoarthritis was noted during 1-year follow-up. The Tönnis grading scale of hip osteoarthritis was less than 2 in all preoperative and postoperative radiographs.

Panel A and B:

A 15-year-old girl with multiple epiphyseal dysplasia. She complained of bilateral intractable hip pain and progressive limp for years. Anterior–posterior pelvic radiograph (panel A) showed severe bilateral hip dysplasia and hip subluxation with broken Shenton line. Frog leg radiograph (panel B) showed hip joints were reducible without hinge abduction.

Panel C:

The staged Bernese PAO was performed for bilateral hip disorders. Postoperative anterior–posterior pelvic radiograph (panel C) showed correction of hip dysplasia with restored Shenton line and the reduced femoral head.

### Clinical results

The hip pain and function were all improved after operation. The improvement of clinical outcomes was assessed by the mean HHS with a significant increase from 69.7 (range from 66 to 76) preoperatively to 86.3 (range from 81 to 91) postoperatively (*p* < 0.05). All patients returned to normal activities of daily living at one year postoperatively. The comparison between Pre-operative and post-operative HHS was shown as Table 3.

**Table 3:** The preoperative and postoperative comparisons of Harris Hip Score in patients with MED following the Bernese PAO

Parameters	Pre-operation		Post-operation		*p* value
	Mean	Range	Mean	Range	
Total score	69.7	66 to 76	86.3	81 to 91	0.02
Pain score	20	20 to 20	30	30 to 30	0.02
Function domain	25	23 to 26	27.3	26 to 30	0.13
Physical exam	10.7	9 to 14	13.7	13 to 14	0.39

### Complications and estimated blood loss

There were no obvious complications after operation, including massive bleeding, surgical site infection, nerve palsy or nonunion observed during follow-up at clinic visits. Post-operative course was smooth and all patients recovered well physically. There was no conversion of Bernese PAO to total hip replacement.

## Discussion

MED is caused by abnormalities in type IX collagen and cartilage oligomeric matrix protein [14]. Most of patients develop deformities of the extremities during childhood. The symptoms including intractable pain and progressive deformities of lower extremity can be noted as early as 2-year-old [15]. Due to disorganized endochondral ossification of the epiphyses of long bones, the articular cartilage lacks underlying osseous support that results in degeneration. Cartilage degeneration is most prominent in weight-bearing joints, especially hip joint [16]. End-stage hip secondary osteoarthritis at a relatively young age is common. However, current results of total hip replacement in patients with MED were discontented.

To date, the treatment guideline of MHD to prevent secondary hip osteoarthritis remains elusive. At present, many studies indicate that this progression is inevitable and suggests only conservative treatments. Kim et al. reported a study of 40 patients with MED involved in hip joints treated with conservative strategies, including weight-bearing restrictions, body weight control and physical therapies. The results revealed some degree of improvement in the hip deformity and function [1]. Nevertheless, the conservative treatment only delays osteoarthritis progression mildly and the training course is time-consuming. Most of all, the daily activity may be restricted in order to reduce the burden of the joint. As time goes by, the hip deformity and femoral head morphology deteriorated without corrections.

Patients with MED may have a wide spectrum of hip joint deformity. Surgical intervention could be considered as a time-efficient treatment of choice for these patients, including proximal osteotomy or PAO. For patients with varus or valgus deformity combined with hip dysplasia, proximal femoral osteotomy can serve as a suitable treatment [17]. Lian et al. reported the surgical outcomes of 2 patients with MED underwent intertrochanteric extension osteotomy. Joint function, coxa vara deformity and femoral head coverage were improved in mid-term follow-up [8]. However, for those patients with inadequate coverage of the femoral head and acetabular change, a rotational osteotomy of the pelvis may be superior to intertrochanteric osteotomy [18]. PAO is still the gold-standard treatment for hip dysplasia. Wyles et al. revealed PAO can unequivocally retard the deterioration in patients with hip dysplasia [19]. Although surgical intervention could not alter the ossification of the epiphysis, emerging evidence indicates that an acetabular correction would improve the prognosis of hip disorders in MED. Sponer et al. reported 12 hips in 11 patients with MED were treated by the Steel triple innominate osteotomy. The mean follow-up time was 2 years with the correction of LCEA angle [7]. However, other radiographic results were not reported. In our study, the outcomes of LCEA, ACEA, AI and femoral head coverage were all improved after operation. We demonstrated that Bernese PAO can correct acetabular and femoral morphology 3-dimensionally. Lian et al. reported 2 patients with MED and severe hip deformity were treated with Dega osteotomy. The mid-term outcomes including functional and radiographic were pleasing. [8] However, the Bernese PAO has some advantages that Steel triple innominate osteotomy and Dega osteotomy could not achieve. On the one hand, those osteotomies and bone graft placement are relatively unstable. Post-operation immobilization would be required for a much longer time. On the other hand, it is difficult to achieve optimal medialization and coverage of the acetabular cup [20].

Among various procedures of PAO, the Bernese PAO is a novel technique that exerts many clinical advantages, e.g., better stability, maximal mobility, preservation of the acetabular blood supply, preservation of the hip abductor musculature, medialization of acetabulum and powerful deformity correction. Furthermore, it could be combined with adjunctive procedures if other hip deformity exists [9]. A recent study pointed out that the Bernese PAO provides a satisfactory surgical approach in the treatment of the hip deformity with global dysplasia in cerebral palsy patients [21]. With the analyses of surgical results for global dysplasia of the acetabulum in our patients, we prove that Bernese PAO is an optimal procedure to correct this rare congenital disorder.

In our studies, the mean operating age of patients was merely 14.3 years, yet plain films revealed that triradiate cartilages were closed. For hip dysplasia, the Bernese PAO is the treatment of choice in our experience for those patients, especially for those with global dysplasia of the acetabulum diagnosed from preoperative 3D-CT scan. To the best of our knowledge, there was no study using Bernese PAO for MED patient with symptomatic hip dysplasia in English literatures before. The short-term outcome is satisfactory. Radiographic parameters including LCEA, ACEA, AI and the femoral head coverage ratio were significantly corrected. Of great importance, the improvement of hip function is in accordance with radiographic correction. The patient could have normal activity of daily living without restriction and major discomforts. No major complication was noted during surgery or follow-up. Because the small incision was performed, the wound pain could be well tolerated under control of medications and the patient could start rehabilitation as early as 1 day post operation. Furthermore, the Bernese PAO provides adequate medialization of hip joint. In case the osteoarthritis progresses, a better joint morphology can offer easier positioning of total hip replacement.

There are limitations to our study. First, the case number is not large due to the study nature of rare disease. Given the low incidence of MED, it is hard to perform a prospective study in a large sample size. Second, it is a short-term follow-up study to investigate the effects of time-efficiency following the surgical treatment. However, our study does provide valuable information regarding the 1-year results for the clinicians facing the management for such difficult cases of congenital skeletal dysplasia. A longer follow-up study is necessary to investigate the long term clinical outcomes of the operated hips.

## Conclusions

The Bernese PAO is a feasible option for treatment of the hip disorders in MED patients, providing the benefits of successful functional recovery without complications. The surgical treatment is effective and efficient through reshaping acetabular and femoral morphology three-dimensionally. In our study, the preliminary results showed the procedure not only improved radiographic outcomes but also hip function. A long-term follow-up in a larger sample size study is warranted in the future.

## Data Availability

The datasets used and/or analyzed during the current study are not openly available due to reasons of sensitivity and are available from the corresponding author upon reasonable request. The data is securely stored at National Taiwan University Hospital. The datasets used and/or analyzed during the current study are available from the corresponding author on reasonable request.

## Abbreviations

ACEA: Anterior center–edge angle
AI: Acetabulum index
HHS: Harris hip score
LCEA: Lateral center–edge angle
MED: Multiple epiphyseal dysplasia
PAO: Periacetabular osteotomy
